# Diagnostic Potential of Combined Skin Morphometric Analysis and Salivary Alpha-Synuclein Oligomers in Parkinson’s Disease

**DOI:** 10.3390/cells15070634

**Published:** 2026-04-01

**Authors:** Claudia Caturano, Francesco Emanuele Bellomi, Eleonora Galosi, Maria Ilenia De Bartolo, Matteo Costanzo, Francesca Arciprete, Maria Zingariello, Massimo Marano, Antonella Conte, Giovanni Fabbrini, Romina Mancinelli, Daniele Belvisi, Andrea Truini, Alfredo Berardelli, Giorgio Vivacqua

**Affiliations:** 1 Laboratory of Microscopic and Ultrastructural Anatomy, Integrated Research Center PRAAB, Campus Bio-Medico University of Rome, 00128 Rome, Italy; claudia.caturano@alcampus.it (C.C.); francesco.bellomi@alcampus.it (F.E.B.); f.arciprete@unicampus.it (F.A.); m.zingariello@unicampus.it (M.Z.); g.vivacqua@unicampus.it (G.V.); 2 Department of Human Neuroscience, Sapienza University of Roma, 00185 Rome, Italy; eleonora.galosi@uniroma1.it (E.G.); matteo.costanzo@uniroma1.it (M.C.); antonella.conte@uniroma1.it (A.C.); giovanni.fabbrini@uniroma1.it (G.F.); daniele.belvisi@uniroma1.it (D.B.); alfredo.berardelli@uniroma1.it (A.B.); 3 Department of Radiological Sciences, Oncology and Anatomical Pathology, Sapienza University of Roma, V.le Delle Università 30, 00161 Rome, Italy; 4 IRCCS Neuromed, 86077 Pozzilli, Italy; 5 Department of Neuroscience, Istituto Superiore di Sanità, 00161 Rome, Italy; 6 Unit of Neurology, Neurophysiology, Neurobiology and Psychiatry, Department of Medicine, Campus Bio-Medico University of Rome, 00128 Rome, Italy; m.marano@policlinicocampus.it; 7 Fondazione Policlinico Universitario Campus Bio-Medico, Viale Alvaro del Portillo 200, 00128 Rome, Italy; 8 Department of Anatomical, Histological, Forensic Medicine and Locomotor Apparatus Sciences, Sapienza University of Roma, 00185 Rome, Italy; romina.mancinelli@uniroma1.it

**Keywords:** alpha-synuclein, skin biopsy, Parkinson’s disease, melanocytes, collagen type IV

## Abstract

Oligomeric species of alpha-synuclein (α-syn) in saliva and phosphorylated α-syn deposits in the skin are established molecular biomarkers for Parkinson’s disease (PD). However, existing research has yet to fully explore the diagnostic potential of non-phosphorylated α-syn and other cutaneous morphometric parameters, such as variations in collagen type IV within the dermis and epidermis or α-syn expression in melanocytes. This study aims to evaluate and compare the diagnostic utility of these skin morphometric parameters in differentiating 32 PD patients from 19 healthy subjects (HSs), while also examining their correlation with salivary α-syn oligomer levels. Skin biopsies were analyzed via immunofluorescence and confocal microscopy, while salivary oligomeric α-syn was quantified through competitive ELISA. Results revealed a significant reduction in α-syn-positive fibres in PD patients compared to HSs (0.91; <0.0001). Conversely, the collagen staining area and the number of α-syn-positive melanocytes were significantly increased in the skin of PD patients. Specifically, the collagen type IV staining area was significantly higher in the dermis and surrounding the sweat glands of PD patients, demonstrating optimal diagnostic power (0.9448; <0.0001). Similarly, the increase in α-syn-positive melanocytes in PD patients showed robust diagnostic potential (0.84; <0.001). Salivary α-syn oligomers accurately discriminated between PD and HS groups. Furthermore, significant correlations were found between collagen type IV and melanocyte morphometric parameters and various clinical scores in PD. Our findings highlight how multimodal morphometric analysis of the skin can enhance diagnostic accuracy in PD, supporting the use of salivary and cutaneous biomarkers as complementary tools that may reflect distinct aspects of PD pathology.

## 1. Introduction

Parkinson’s disease (PD) is a progressive neurodegenerative disorder characterized by motor symptoms including bradykinesia, rigidity, tremor, and postural instability [1,2] largely attributed to the degeneration of dopaminergic neurons in the substantia nigra. PD is also associated with several debilitating non-motor symptoms, including autonomic dysfunction, sleep disorders, and cognitive impairment, which often precede motor symptom onset [1,3,4] and are largely driven by the accumulation of misfolded alpha-synuclein (α-syn) aggregates in the central nervous system (CNS) and in various anatomical regions throughout the body, including enteric neurons [5,6], skin nervous fibres [7,8,9] or minor salivary glands [10].

The diagnosis of PD currently relies on clinical assessment, which can be challenging, particularly in the early stages or in the differential diagnosis with atypical parkinsonisms [11]. The lack of reliable molecular biomarkers hinders timely intervention and the development of effective disease-modifying therapies.

Early studies have demonstrated the presence of α -syn in the CSF [12] of PD patients, but there is now a need to identify accessible and minimally invasive biomarkers that can aid in the early PD diagnosis and biological definition of the disease. In this scenario, oligomeric α-syn in saliva [13,14,15] and phosphorylated α-syn deposits in nerve fibres within the skin [16,17,18] have shown considerable promise.

In a previous study [19], investigating the relationship between skin deposits of phosphorylated α-syn and the levels of oligomeric α-syn in saliva, we demonstrated that they are not directly correlated. Morphological parameters detectable in PD skin biopsies go beyond phosphorylated α-syn. Melanocytes share common biochemical and morphological features with dopaminergic neurons affected in PD [20,21]. Previous studies have highlighted the increased prevalence of melanomas in patients with PD [22,23,24,25] and the involvement of α-syn in the regulation of melanin biosynthesis [26,27,28,29]. Collagen and extracellular matrix are also altered in PD [30]. Polymorphism of collagen VI has been related to increased risk of developing PD [31], while α-syn has been reported to dysregulate COL4A2 gene expression, impairing the function of the ER–Golgi network and thus exacerbating protein misfolding and aggregation [32]. However, there are no studies examining these latter morphometric parameters in PD patients in comparison to healthy subjects.

In the present study, we therefore evaluated the expression and aggregation of α-syn in melanocytes and the variations in collagen type IV architecture in dermis and epidermis. Moreover, to investigate whether alterations in collagen type IV and melanocyte α-syn correlate with α-syn pathology in the skin nerve fibres and in saliva, we also investigated cutaneous non-phosphorylated α-syn and α-syn oligomers in saliva. Finally, we employed correlation analyses to assess whether morphometric changes in the skin correlate with clinical scores of PD patients.

## 2. Materials and Methods

### 2.1. Study Participants and Clinical Assessment

Thirty-two patients with Parkinson’s disease (PD) were consecutively enrolled at the outpatient clinic of the Department of Human Neurosciences, Sapienza University of Rome. Diagnoses were established in accordance with the International Parkinson and Movement Disorder Society diagnostic criteria [28]. We also recruited a control group of 19 healthy subjects (HSs), consisting of healthy volunteers and non-consanguineous caregivers of the PD patients. To rule out any concomitant neurological conditions, the control subjects underwent a thorough preliminary assessment, featuring a comprehensive medical history and a detailed neurological examination. All participants provided written informed consent. The study protocol was approved by the ethical committee of the Policlinico Umberto I Hospital (n. 5433, prot n. 548/19) and was conducted in strict adherence to the Declaration of Helsinki. For all patients, the neurological assessment involved disease staging using the Hoehn and Yahr scale (H&Y). Motor symptom severity was quantified using the Italian version of the Movement Disorder Society-sponsored revision of the Unified Parkinson’s Disease Rating Scale (MDS-UPDRS) Part III, whereas motor complications—including off periods and dyskinesias—were assessed via the MDS-UPDRS Part IV. The severity of non-motor symptoms was measured using the Non-Motor Symptoms Scale for PD (NMSS). Lastly, current pharmacological treatments were recorded, allowing the levodopa equivalent daily dose (LEDD) to be calculated for each patient (Table 1).

To assess phosphorylated α-synuclein aggregates in melanocytes, cervical (C7-level) skin biopsies were obtained from five patients with Parkinson’s disease (PD) recruited at Policlinico Campus Biomedico, as a subset of the main PD cohort.

### 2.2. Saliva Sample Collection and ELISA Analysis

Unstimulated whole saliva samples were collected from all participants in accordance with standardized protocols [14,29]. Briefly, anamnestic data concerning both oral and systemic health status were obtained for each participant prior to collection. Subjects were required to abstain from eating, drinking, and smoking for 2 h, and to avoid alcohol consumption for 12 h before providing the sample; they also performed a self-washing of the oral cavity 1 h before sample collection. The sample collection was preceded by an inspection of the oral cavity to rule out any existing pathologies. To remove cellular debris, the saliva samples were immediately centrifuged at 5000× *g* and 4 °C. The samples were then treated with a protease inhibitor cocktail (Sigma-Aldrich, P2714) and stored in low-bind 1.5 mL Eppendorf tubes at −80 °C until ELISA analysis. Before proceeding with the ELISA, the total protein concentration of each saliva sample was determined using a BCA Protein Assay Kit (Thermofisher Scientific, Heysham, UK), which ensured the normalization of protein levels across samples from PD patients and HSs using 0.001 M phosphate-buffered saline (PBS). Salivary oligomeric α-syn levels were quantified using a human α-syn Competitive Oligomer ELISA Kit (MyBioSource, San Diego, CA, USA, MBS730762), consistent with previous reports [9,30]. Spectrometric analysis at a 450 nm wavelength, performed with an appropriate microplate reader (LT 4000, Labtech, Heathfield, Tonbridge, UK), was employed to determine the salivary concentration of α-syn oligomers. Standard curves were generated by correlating the optical density measurements of the standards with their respective concentrations.

### 2.3. Skin Biopsy and Immunofluorescence

Both PD patients and HSs underwent a skin biopsy at a cervical site (C7 level), located 2 cm laterally to the dorsal spinous line. As previously reported [31], the procedure was performed using a 3 mm disposable circular punch following local anesthesia with lidocaine. Biopsies were fixed for 24 h at 4 °C in Zamboni’s fixative and subsequently cryoprotected overnight. Sectioning was conducted at −23 °C using a cryostat (MEV, SLEE medical, Nieder-Olm, Germany) to obtain 10 μm thick sections. For each participant, four 10 μm thick sections were selected through random sampling of the skin biopsy for analysis. These sections were blocked with 5% normal donkey serum for 1 h and immunostained overnight with a monoclonal mouse primary antibody for α-syn (BD Biosciences, San Diego, CA, USA; 610787, 1:500) and a polyclonal rabbit antibody for Collagen IV (Abcam, Cambridge, UK; ab138492, 1:750). After washing, the sections were incubated for 2 h with anti-mouse-488 (Jakson, West Grove, PA, USA; 715-545-150, 1:200) and anti-rabbit-555 (Jakson, West Grove, PA, USA; 711-165-152, 1:200) secondary antibodies. To investigate phosphorylated α-syn aggregates within melanocytes, 40 μm thick skin sections were stained with a rabbit polyclonal antibody for pS129 α-syn (Abcam, Cambridge, UK; ab51253, 1:1000) and a mouse monoclonal antibody for MelanA (Abcam, Cambridge, UK; ab187369, 1:500), followed by a 2 h incubation with the same secondary antibodies (anti-mouse-488 and anti-rabbit-555). DAPI was added for nuclear counterstaining prior to coverslipping.

### 2.4. Image Acquisition and Morphometric Analysis

Fluorescent images were acquired using a confocal Eclipse Ni microscope equipped with specific filter cubes to identify the employed fluorochromes. Digital image recording was performed with a Nikon DS-Qi1Nc camera and NIS Elements software (version BR 4.20.01). A Z-stack of each section at 20× with an interval of 0.5 μm for each layer of the *Z*-axis and a Maxi-P image based on the Z-stack was obtained from each sample. Standardized image acquisition parameters were maintained across all samples (including time of exposure, magnification, and LUTS). The analysis was carried out independently by two blinded operators with expertise in immunofluorescence analysis (CC, FEB). Colocalization with PGP9.5 immunostaining was used to identify the nerve fibres positive for α-syn. Melanocytes were morphologically identified as the cells at the border between dermis and epidermis, while collagen IV staining was investigated at both epidermis and dermis and around adnexal structures. Quantitative morphometric analysis was performed using the specialized image analysis software ImageJ (version 1.53t) (National Institutes of Health http://imagej.net/ij (accessed on 25 March 2026)). The average epidermal thickness was estimated. Custom MACROs were developed and employed to perform the analysis on the selected images. Epidermis, dermis, as well as adnexal structures, including nerve endings, sweat glands or vascular structures, were histologically identified by histology experts to define the ROI. Dermal and epidermic areas of equivalent dimension have been analyzed from each skin biopsy. Fluorescence quantification for both α-syn and collagen IV was conducted by applying different thresholds to highlight and quantify staining at different degrees of intensity; these thresholds were maintained consistently across all analyzed sections. The extracted data for collagen VI staining include: the staining area, the average intensity above each threshold and the percentage of positively stained area relative to the total area under analysis. Nerve fibres positive for monomeric α-syn were quantified in number and percentage relative to the total area analyzed. Melanocytes were morphologically identified as cells with a diameter ranging between 20 and 70 μm, a dendritic shape and a typical localization at the dermo-epidermal junction. α-syn-positive melanocytes were quantified in number and fluorescence intensity, as well as the percentage of positive area in relation to the total area analyzed. For the detection of pS129 α-syn, melanocytes were identified with specific MelanA staining and acquired at high magnification (60×) with the confocal microscope. For each parameter, the value for everyone represented a mean of the four skin sections analyzed. All analyses were performed by researchers blinded to the clinical status of the participants to minimize bias.

### 2.5. Statistical Analysis

Data were analyzed using GraphPad software version 10 (Prism, USA). Data distribution was assessed for normality using the Anderson-Darling, Shapiro–Wilk, and Kolmogorov–Smirnov tests, with a threshold value of *p* = 0.05. Comparisons between PD patients and healthy subjects for salivary α-syn levels and skin morphometric parameters were performed using non-parametric tests (e.g., two-tailed T-test with Welch’s correction for salivary α-syn oligomers and Mann–Whitney U test for skin morphometric parameters). Receiver operating characteristic (ROC) curve analysis was used to evaluate the diagnostic accuracy of individual biomarkers, with the area under the curve (AUC) reported as a measure of diagnostic performance. Correlations between salivary oligomeric α-syn and skin morphometric parameters were assessed using Spearman’s rank correlation coefficient. Similarly, correlations between the different biomarkers and clinical scores of the PD cohort were assessed, and a correlation matrix based on multiple correlation was obtained. A *p*-value of less than 0.05 was considered statistically significant.

## 3. Results

### 3.1. Skin Morphometric Parameters in Parkinson’s Disease

**Figure 1:**
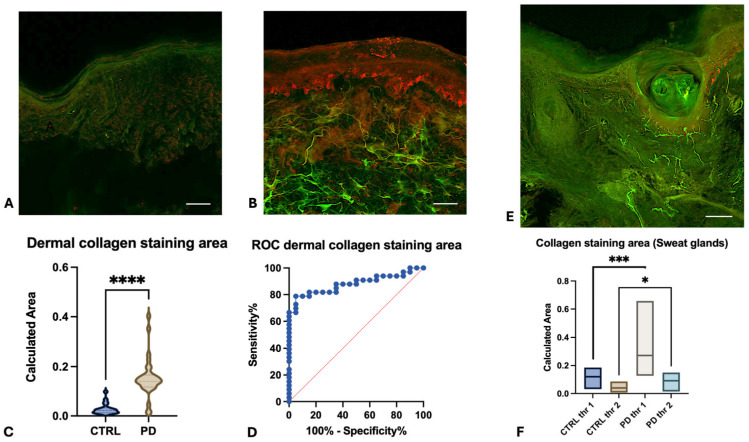
Double immunofluorescence staining for collagen type IV (Alexa-fluo 488—green) and α-syn (Alexa-fluo 568—red) in healthy subjects (**A**), and PD patients (**B**). A robust increase in collagen staining area is detected in the dermis of PD patients with respect to healthy subjects (*p* < 0.0001, Mann–Whitney U 32—(**C**)). *ROC* analysis discloses an optimal AUC of 0.9448 in differentiating PD from healthy subjects (**D**). A particular increase in collagen-stained area is demonstrated around the sweat glands, as shown in (**E**,**F**) (*p* 0.0147, Mann–Whitney U 18). Linear bar 60 μm. *p* value legend―****: <0.0001, ***: <0.001, *: <0.1.

**Figure 2:**
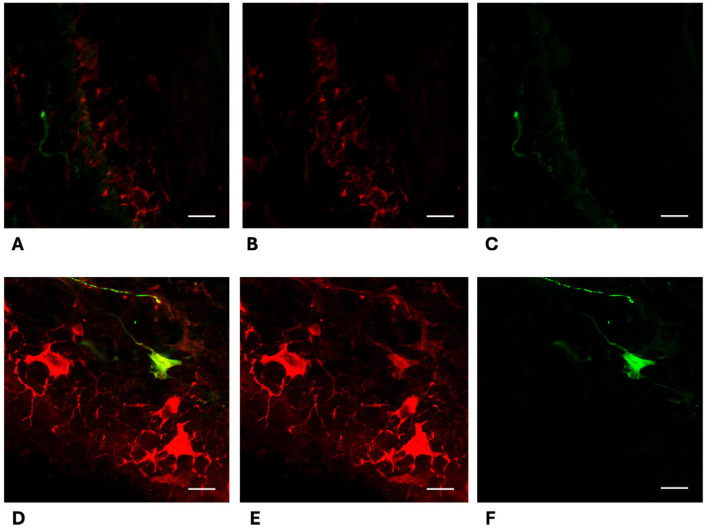
Double immunofluorescence staining for collagen type IV (Alexa-fluo 488—green) and native α-syn (Alexa-fluo 568—red) of the dermo-epidermal junction in healthy controls (**A**–**C**) and PD patients (**D**–**F**). In PD patients, melanocytes (detected as pyramidal-shaped cells with dendritic branches) appear hypertrophic with an increased dendritic branching and a massive α-syn staining in the cytoplasm, while only a few α-syn positive spots are detected in melanocytes from control subjects. Linear bar 20 μm.

**Figure 3:**
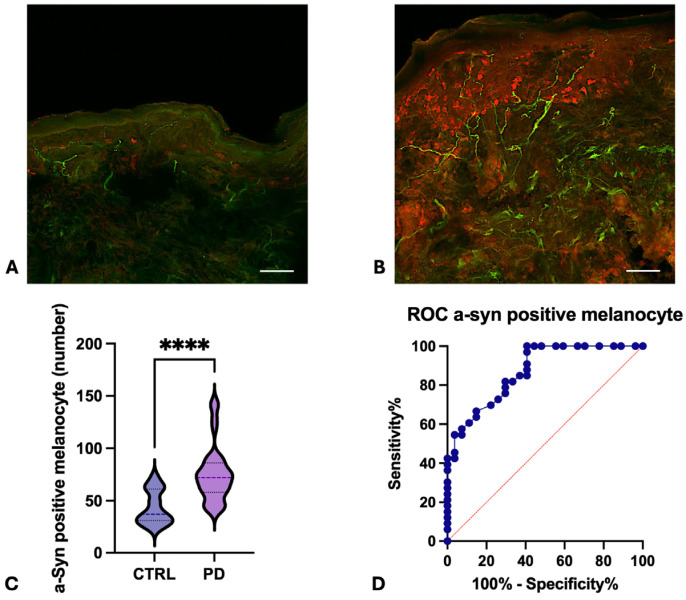
Double immunofluorescence staining for collagen type IV (Alexa-fluo 488—green) and α-syn (Alexa-fluo 568—red) in healthy subjects (**A**) and PD patients (**B**). A robust increase in the number of α-syn-positive melanocytes is detected at the border between dermis and epidermis of PD patients with respect to healthy subjects (*p* < 0.0001, Mann–Whitney U 115.5—(**C**)). *ROC* analysis discloses a suboptimal AUC of 0.84 (**D**). Linear bar 60 μm. *p* value legend―****: <0.0001.

**Figure 4:**
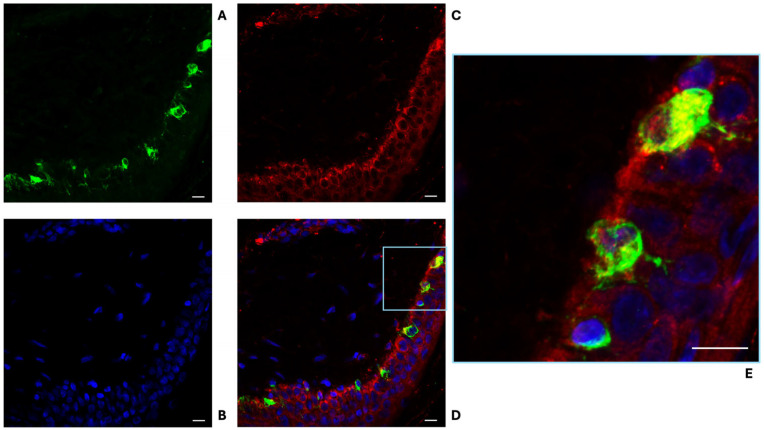
Immunofluorescence staining for MelanA (Alexa-fluo 488—green) (**A**), DAPI (**B**) (blue), and pS129 α-syn (Alexa-fluo 568—red) (**C**) in the skin of PD patients. Merge figure (**D**), detailed at high magnification in (**E**), demonstrates the presence of pS129 α-syn aggregates in melanocytes as colocalization with MelanA (yellow). Linear bar 20 μm in (**A**–**D**), 60 μm in (**E**).

**Figure 5:**
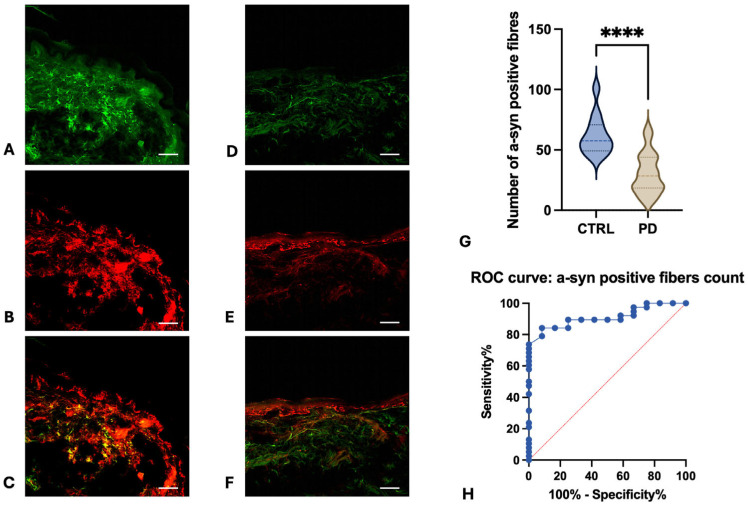
Double immunofluorescence staining for PGP 9.5 (Alexa-fluo 488—green. (**A**): Healthy subjects and (**D**): PD) and α-syn (Alexa-fluo 568—red. (**B**): Healthy subjects and (**E**): PD). A dramatic reduction in nerve fibres positive for α-syn is detected in the dermis and the epidermis of PD patients with respect to healthy subjects (*p* < 0.0001, Mann–Whitney U 21—(**G**)), as demonstrated by merge image ((**C**): healthy subjects and (**F**): PD). *ROC* analysis discloses an AUC of 0.91 in differentiating PD from healthy subjects (**H**). Linear bar 60 μm. *p* value legend―****: <0.0001.

Morphometric analysis of skin biopsies revealed several significant differences between PD patients and healthy subjects:

- Collagen Type IV Staining Area. The area stained by collagen type IV was quantified in three different sections for each participant. Both the overall collagen IV-stained area in the dermis and specific areas of collagen aggregation around sweat glands were significantly increased in the skin of PD patients in comparison to healthy subjects (Figure 1A,B). The average overall collagen-stained area was 0.1401 in the dermis of PD patients and 0.02239 in the dermis of healthy subjects (Figure 1C, *p* < 0.0001). Notably, the collagen intensity was particularly higher around sweat glands, where it appears organized in thick and condensed fibres (Figure 1E,F:—*p* < 0.001 for threshold 1 and <0.1 for threshold 2). This morphometric parameter demonstrated an optimal diagnostic power in differentiating PD patients from healthy subjects, achieving an AUC of 0.9448 (*p* < 0.0001) and highlighting the substantial changes in dermal collagen architecture in PD (Figure 1D). Conversely, epidermal collagen type IV staining was not significantly different between PD and healthy subjects.
- α-Syn-Positive Melanocytes. The number of α-syn-positive melanocytes was investigated in three skin sections for each participant. α-syn-positive melanocytes were detected at the border between dermis and epidermis, with the typical dendritic shape. Intensity of α-syn staining was higher in melanocytes from PD patients (Figure 2D–F) in comparison to healthy subjects (Figure 2A–C). The averaged calculated number of α-syn-positive melanocytes per stained area was 37 in healthy subjects and 72 in PD patients (*p* < 0.0001—Figure 3C). The increasing of α-syn-positive melanocytes also showed a promising diagnostic potential, with an AUC of 0.84 (*p* < 0.001) in differentiating PD patients from healthy subjects (Figure 3D). While less robust than the collagen type IV-stained area, this morphometric parameter highlights the involvement of melanocytes in synucleinopathy. The presence of pS129- α-syn aggregates was detected in the melanocytes of 2 PD patients, among the five investigated (Figure 4A–D), while in three of them, phosphorylated α-syn was absent from MelanA-positive cells, confirming the established localization within the autonomic nerve fibres.
- α-Syn-Positive Fibres. To detect the concomitant occurrence of α-syn pathology, α-syn-positive nerve fibres were quantified for number and staining intensity in three different sections for each subject, considering both dermis and epidermis. A significant reduction in the number of α-syn-positive fibres was observed in the skin of PD patients compared to healthy subjects (Figure 5A–F). The average number of fibres per stained area detected in healthy subjects was 61.92, with respect to 30.34 detected in PD patients (*p* < 0.0001—Figure 5G). This loss of α-syn-positive fibres yielded a diagnostic potential with an AUC of 0.91 (*p* < 0.0001) (Figure 5H). However, the intensity of α-syn-positive fibres was increased in PD patients in comparison to healthy subjects, indicating that, although reduced in number, the nerve fibres in PD skin contain aggregated α-syn, which is reflected by the higher staining intensity.

### 3.2. Salivary α-Synuclein Oligomers as a Diagnostic Biomarker

**Figure 6:**
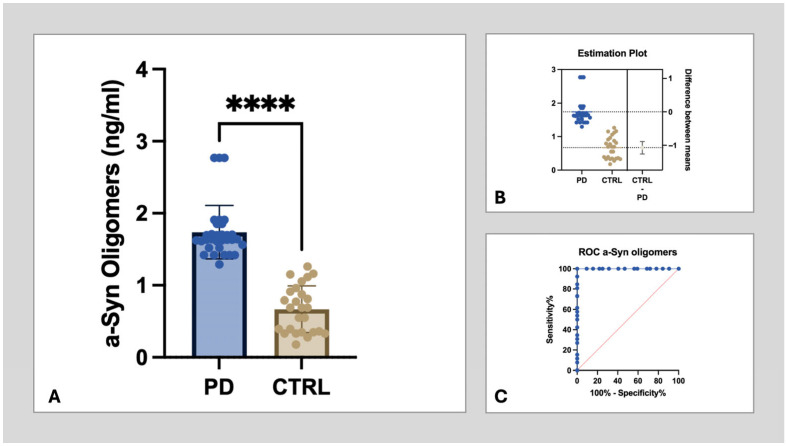
Concentration of α-syn oligomers in the saliva of PD patients and healthy controls (CTRL) as measured by ELISA (**A**). A significant increase in the concentration of oligomeric α-syn is detectable in PD in comparison to CTRL (*p* < 0.0001, Welch-corrected t 11.74), as also demonstrated by the estimation plot (difference by means −1.071, R-squared 0.7124—(**B**)). *ROC* analysis discloses an AUC of 0.98, supporting a high diagnostic accuracy (**C**). *p* value legend―****: <0.0001.

Consistent with previous studies, salivary oligomeric α-syn levels were dramatically increased in the saliva of PD patients in comparison to healthy subjects (Figure 6A,B). The average oligomeric α-syn in PD saliva was 1.739 ng/mL in PD patients, while it was 0.6674 ng/mL in healthy subjects. Receiver operating coefficient (ROC) analysis revealed, in fact, an AUC of 0.99 (*p* < 0.0001) in differentiating PD from healthy subjects (Figure 6C).

### 3.3. Correlations Between Salivary and Skin Biomarkers

**Figure 7:**
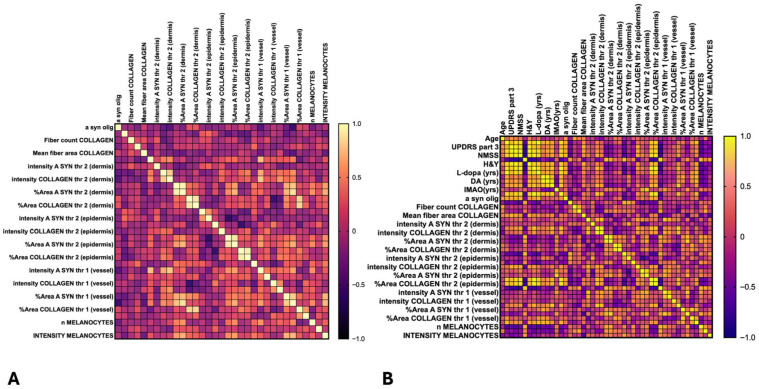
Heatmap reporting the correlations assessed by Spearman-rank correlation coefficient between salivary α-syn oligomers and the different skin morphometric parameters (**A**) and between both salivary α-syn oligomers and skin morphometric parameters with the principal clinical scores of the PD cohort (**B**).

Spearman-rank correlation analysis with a correlation matrix was used to correlate the different skin morphometric parameters with salivary α-syn oligomers (Figure 7A).

As expected, the different morphometric measures for each biological parameter (collagen type IV staining, α-syn-positive melanocytes, monomeric α-syn staining) were significantly correlated between them, while a significative negative cross-correlation was detected only for the intensity of α-syn staining in the dermis and the intensity of collagen staining in the same area considering both threshold 1 (*p* 0.004) and threshold 2 (*p* 0.001).

A robust negative correlation was detected between salivary concentration of α-syn oligomers and the area of α-syn-positive fibres in both dermis (*p* 0.005) and around the blood vessels (*p* 0.004), suggesting that an increased systemic α-syn pathology is associated with a more severe peripheral neuropathy, detected with the loss of α-syn-positive nerve fibres.

### 3.4. Correlations of Salivary and Skin Biomarkers with Clinical and Therapeutic Scores

Spearman-rank correlation coefficient and correlation matrix were also employed to correlate skin morphometric parameters and salivary α-syn oligomers with the main clinical scores of our cohort of PD patients, including: MDS-UPDRS part 3 to assess the motor performance, the NMSS to assess nonmotor symptoms, the MOCA score to assess cognitive performance, and the calculated LEDDs, assessing the dopaminergic treatment (Figure 7B).

A robust positive correlation has been found between MDS-UPDRS part 3 (*p* 0.001), total UPDRS (*p* < 0.0001), NMSS (*p* < 0.0001) and the collagen type IV-stained area in both dermis and epidermis, as well as the epidermic thickness, suggesting that skin collagen deposition correlates with disease severity. As expected, a robust negative correlation has been found between α-syn-positive fibres count and the different clinical scores: MDS-UPDRS part 3 (*p* < 0.05), total MDS-UPDRS (*p* < 0.05), NMSS (*p* < 0.005), and MOCA (*p* < 0.005). Finally, the number of α-syn-positive melanocytes and the area of α-syn stained melanocytes negatively correlated with the daily dose of levo-DOPA (*p* < 0.04) and with the duration of levo-DOPA treatment (*p* < 0.05), supporting the involvement of DA therapy in the biology of melanocytes.

Finally, in this PD cohort, a robust positive correlation was detected between salivary α-syn oligomers concentration and the main clinical scores: MDS-UPDRS part 3 (*p* < 0.0001), NMSS (*p* < 0.0001), MOCA (*p* 0.0005) and H&Y (*p* 0.0005).

## 4. Discussion

In the present study, we have analyzed the architectural changes in collagen type IV and the expression of α-syn in melanocytes in the skin of PD patients, in comparison to healthy subjects. To evaluate the correlation of these morphometric parameters with α-syn pathology, we have also investigated the expression of non-phosphorylated α-syn in the skin nerve fibres and the concentration of α-syn oligomers in saliva. As a novel finding, we demonstrate that collagen IV architectural changes, particularly in the dermis and around sweat glands, and α-syn overexpression in melanocytes are signature pathological features in PD patients and disclose a promising diagnostic potential. Moreover, we reveal a negative correlation between salivary α-syn oligomers and α-syn-positive fibres in the skin of PD patients and a direct correlation between different scores of disease severity and both loss of α-syn-positive fibres and an increase in collagen deposition in the skin. Finally, we show an inverse correlation between α-syn intracellular amount in melanocytes and levo-DOPA therapy as measured by LEDDs.

The most striking finding from our skin biopsy analysis is the significant increase in collagen IV staining area in PD patients, particularly in the dermis and around sweat glands. This skin morphometric parameter demonstrated the highest diagnostic accuracy in comparison to healthy subjects, suggesting that alterations in collagen architecture may possibly be a potential complementary biomarker for PD. The peri-adnexal regions, rich in autonomic innervation, may be particularly affected by α-syn-related pathology, leading to reactive collagen deposition or altered collagen turnover. Immunomodulatory effects of the autonomic nervous fibres have been linked to collagen regulation in different autoimmune pathologies [32,33,34]. Chronic inflammation, oxidative stress, or direct α-syn pathology within cutaneous nerve fibres could contribute to the alterations in the extracellular matrix and the dysregulation of collagen deposition in the dermis and at peri-adnexal regions, explaining the increased collagen staining we report in PD patients.

The increase in α-syn-positive melanocytes in PD patients, although showing a sub-optimal diagnostic potential (AUC 0.84), is an intriguing finding. Melanocytes are neuroectodermal in origin and share developmental pathways with neurons. The presence of α-syn aggregates within these cells has also been sustained by the detection of pS129 α-syn species and suggests a broader involvement of α-syn pathology in neuroectodermal cells beyond the nervous system, potentially reflecting systemic vulnerability of Tyrosine Hydroxylase-synthesizing cells to the formation of α-syn aggregates. In accordance, a crosstalk between amyloid α-syn fibrils and the functional amyloid pre-melanosomal protein (Pmel17) has been observed in melanoma cells [18], where α-syn regulates melanin biosynthesis [35,36,37], by interacting with tyrosinase in a way similar to that occurring in DA neurons [20,38,39]. Other evidence has reported that neuromelanin enhances α-syn aggregation in DA neurons [40,41], and this pathological mechanism can also occur in melanocytes. This finding opens new avenues for exploring the application of melanocytes as a peripheral cellular model to investigate α-syn pathology in PD.

The significant reduction in nerve fibres positive for monomeric α-syn in the skin of PD patients supports the concomitance of α-syn pathology with the alteration of other skin morphometric parameters. This finding may apparently be in contrast with the presence of phosphorylated α-syn aggregates and could reflect either the reduction in monomeric α-syn in the skin nerve fibres due to the aggregates’ formation, as the loss of autonomic and somatosensory fibres affected by synucleinopathy, as previously reported in different PD cohorts [42,43,44]. This phenomenon, often termed “denervation,” has also been observed in other neurodegenerative conditions, including amyotrophic lateral sclerosis or Alzheimer’s disease [38,45], and could represent an earlier or distinct pathological process, besides the formation of phosphorylated α-syn aggregates. In accordance, a compelling number of studies support small fibre peripheral neuropathy as the anatomical substrate of neuropathic pain and dysautonomia in PD [39,44,46,47]. Interestingly, the loss of nerve fibres positive for monomeric α-syn was directly associated with a severe clinical phenotype and with an increased dopaminergic medicalization. These findings might be explained either by a direct correlation between the degree of small fibre neuropathy and the disease severity [48,49], as with the occurrence of peripheral neuropathy due to vitamin B deficiency in the course of dopaminergic therapy [50,51]. Further studies in larger cohorts are needed to confirm the diagnostic power and the predictive value of peripheral nerve fibre loss on disease progression.

The accuracy of salivary α-syn oligomers as a diagnostic biomarker in PD aligns with a growing body of evidence, emphasizing its accessibility and non-invasive nature as well as its correlation with disease severity [13,14,15]. The inverse correlation between the loss of nerve fibres positive for α-syn in the skin and the concentration of salivary α-syn oligomers suggests that peripheral neuropathy is much more severe in the case of enhanced systemic α-syn pathology, independently of the formation of skin aggregates of phosphorylated α-syn [9]. The overall correlation analysis with the other skin morphometric parameters investigated confirms that salivary and skin biomarkers provide complementary information, underscoring the complex and heterogeneous nature of PD pathology [9]. Salivary α-syn oligomers may reflect systemic or central nervous system α-syn aggregation [15], while the skin parameters capture peripheral nervous system involvement, autonomic dysfunction, or even unique aspects of α-syn pathology within non-neuronal cells. On the other hand, the robust positive correlation detected between salivary α-syn oligomers and the clinical scores in the present cohort of PD patients is probably in relation to the heterogeneity of disease severity and disease duration of the patients enrolled and confirms the results reported in previous cohorts with similar clinical features [52].

Another interesting finding is the inverse correlation between the intracellular amount of α-syn in melanocytes and the amount of dopaminergic therapy measured by LEEDs. Levodopa is a biological modulator for the biosynthesis of melanin [53], and previous studies have also demonstrated the increased proliferation of melanocytes in PD patients treated with levodopa therapy [54,55]. The relation of melanocytes α-syn with dopaminergic therapy has not been investigated yet. However, α-syn also modulates melanin biosynthesis [35,37], suggesting that α-syn and levodopa could have a complementary effect on the metabolism of melanocytes and that dopaminergic therapy may reduce the overexpression and the accumulation of α-syn in melanocytes.

A consistent limitation of the present study includes the small sample size, which may undermine the significance and the diagnostic potential of our morphometric analyses. Moreover, although the reported specificity of the method employed for the detection of α-syn oligomers in saliva [52], it requires additional refinements to prevent possible residual detection of monomeric forms. The small sample size is an intrinsic limitation for the ROC analysis, leading to a possible overestimation of the diagnostic potential. Future studies with larger cohorts are warranted to validate these findings, further investigate the underlying biological mechanisms, and explore the utility of these biomarkers in a real clinical scenario and in understanding novel pathogenetic mechanisms of PD.

## Figures and Tables

**Table 1:** Clinical and demographic characteristics of PD patients and healthy subjects.

Variable	PD Patients (*n* = 32)	Healthy Subjects (*n* = 19)
Age (mean ± SD)	73.1 ± 7.4	57.5 ± 15.9
Sex	11F, 22M	8F, 11M
Age at onset (mean ± SD)	62.9 ± 7.8	-
Disease duration (mean ± SD)	5.1 years ± 4.2	-
MDS-UPDRS Part I (mean ± SD)	8.7 ± 6.48	-
MDS-UPDRS Part II (mean ± SD)	7.7 ± 7.99	-
MDS-UPDRS Part III (mean ± SD)	22.6 ± 13.7	-
MDS-UPDRS Part IV (mean ± SD)	1.66 ± 3.7	-
NMSS total score (mean ± SD)	45.16 ± 35.87	-
MoCA (mean ± SD)	24.59 ± 2.83	-
H&Y (mean ± SD)	1.78 ± 0.66	-
LEDDs (mean ± SD)	427.3 ± 247.4	-

Data from different clinical scores are expressed as means and standard deviations (SD). Categorical variables are expressed as the number of subjects (n). MDS-UPDRS: Movement Disorder Society-sponsored revision of the Unified Parkinson’s Disease Rating Scale; NMSS: Non-motor symptoms scale for PD; MoCA: Montreal Cognitive Assessment; H&Y: Hoehn and Yahr scale; LEDDs: levodopa equivalent daily dose.

## Data Availability

The data presented in this study are available on reasonable request from the corresponding author, due to the presence of ethical restriction on patients clinical scores.
